# Optimization of Cyanocobalamin (Vitamin B_12_) Sorption onto Mesoporous Superparamagnetic Iron Oxide Nanoparticles

**DOI:** 10.3390/molecules29092094

**Published:** 2024-05-01

**Authors:** Jolanta Flieger, Natalia Żuk, Sylwia Pasieczna-Patkowska, Michał Flieger, Rafał Panek, Tomasz Klepka, Wojciech Franus

**Affiliations:** 1 Department of Analytical Chemistry, Medical University of Lublin, Chodźki 4A, 20-093 Lublin, Poland; natalia.zuk@umlub.pl; 2 Department of Chemical Technology, Faculty of Chemistry, Maria Curie Skłodowska University, Pl. Maria Curie-Skłodowskiej 3, 20-031 Lublin, Poland; sylwia.pasieczna-patkowska@mail.umcs.pl; 3 Department of Forensic Medicine, Medical University of Lublin, ul. Jaczewskiego 8b, 20-090 Lublin, Poland; michalflieeeger@gmail.com; 4 Department of Geotechnics, Civil Engineering and Architecture Faculty, Lublin University of Technology, Nadbystrzycka 40, 20-618 Lublin, Poland; rapanek@gmail.com (R.P.); w.franus@pollub.pl (W.F.); 5 Department of Technology and Polymer Processing, Faculty of Mechanical Engineering, Lublin University of Technology, Nadbystrzycka 36, 20-618 Lublin, Poland; t.klepka@pollub.pl

**Keywords:** iron oxide nanoparticles, mesoporous materials, magnetic solid phase extraction, vitamin B12

## Abstract

The techniques used to detect and quantify cyanocobalamin (vitamin B12) vary considerably in terms of detection sensitivity, from the most sensitive, based on radioisotopes and mass spectrometry (MS) with limits of detection (LOD) in fg mL^−1^, to fluorescence (FL) and surface plasmon resonance (SPR) biosensors with LOD values in the range of a few µg mL^−1^. For accurate quantification of an analyte present at trace levels in complex biological matrices, a selective separation and enrichment step is required to overcome matrix interferences and ensure sufficient detection sensitivity. In this study, iron oxide magnetic nanoparticles (IONPs) were used for the extraction and initial preconcentration of cyanocobalamin (vitamin B12). In the dependence of the magnetization on the H-field (hysteresis loop), no coercivity and remanence values were found at 300 K, indicating the superparamagnetic properties of the tested IONPs. Perfluorinated acids were used as amphiphilic agents to allow the sorption of cyanocobalamin onto the IONPs. FT-IR/ATR spectroscopy was used to confirm the sorption of cyanocobalamin on the IONPs. The influence of the addition of a homologous series of perfluorinated acids such as trifluoroacetic acid (TFAA), heptafluorobutyric acid (HFBA), and trichloroacetic acid (TCAA) to the extraction mixture was tested considering their type, mass, and time required for effective sorption. The adsorption kinetics and isotherm, described by the Freundlich and Langmuir equations, were analyzed. The maximum adsorption capacity (*q_m_*) exceeded 6 mg g^−1^ and was 8.9 mg g^−1^ and 7.7 mg g^−1^ for HFBA and TCAA, respectively, as the most efficient additives. After the desorption process using aqueous KH_2_PO_4_ solution, the sample was finally analyzed spectrophotometrically and chromatographically. The IONP-based method was successfully applied for the isolation of cyanocobalamin from human urine samples. The results showed that the developed approach is simple, cheap, accurate, and efficient for the determination of traces of cyanocobalamin in biological matrices.

## 1. Introduction

**Figure 1:**
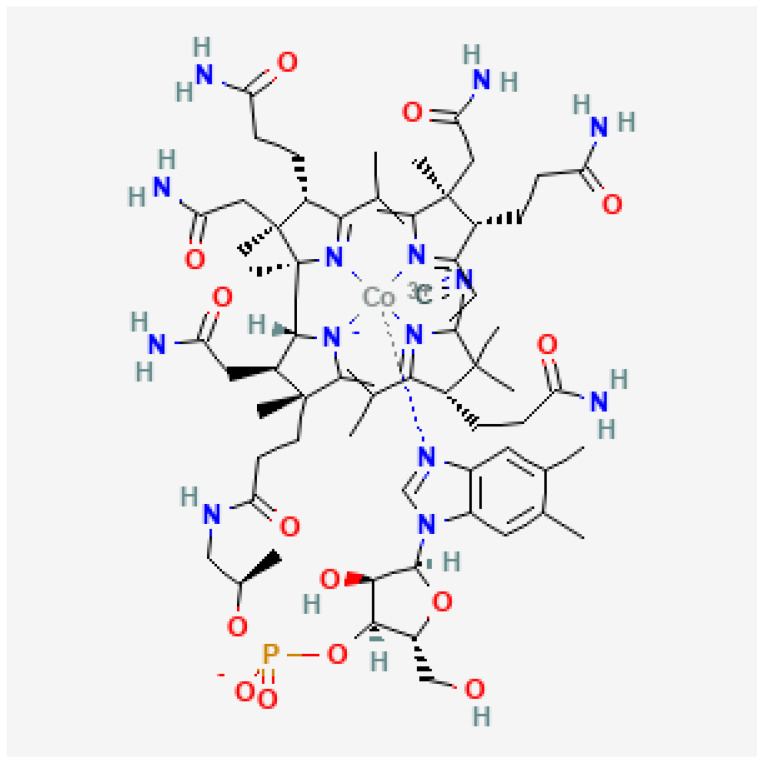
Chemical structure of cyanocobalamin [1].

Vitamin B12 (corrinoids) is a tetrapyrrole complex with a cobalt atom at the centre (Figure 1).

The central cobalt cation has an oxidation state of +3. Vitamin B12 has four basic chemical forms: cyano, hydroxy, methyl and deoxyadenosylcobalamin. In the natural environment, vitamin B12 occurs as deoxyadenosylcobalamin and methylcobalamin. Both forms are very unstable in the presence of light [2]. A stable form of vitamin B12 that does not occur naturally is cyanocobalamin [3]. Vitamin B12 is involved in many physiological processes, including the formation and development of the myelin sheath, red blood cell maturation, and nucleic acid synthesis [4,5,6,7]. Adequate vitamin B12 can be obtained from a well-selected diet rich in animal products (veal, beef, poultry, fish, seafood, eggs, and dairy products) [8,9]. The typical Western diet provides 4 micrograms per day, which is the amount recommended by the European Union for adults [10,11]. However, it should be remembered that vitamin B12 has a rather poor bioavailability, which determines its biological activity. Thus, even with a sufficient intake, only 50% of the B12 is utilized. The causes of vitamin B12 deficiency are poor diet, but also various absorption disorders (e.g., ileal resection, small bowel disease) [12]. Vitamin B12 deficiency can also be caused by a deficiency of intrinsic factor (IF), which is produced by the gastric mucosa (gastrectomy, gastric atrophy). It is estimated that about 15% of the population is vitamin B12 deficient [13,14].

Although the technology for the chemical synthesis of vitamin B12 is known, the multi-step process and economic aspects mean that it is not used on an industrial scale. Cobalamin is currently produced by fermentation using *Aerobacter*, *Azotobacter*, *Bacillus*, *Clostridum*, *Corynebacterium*, *Flavobacterium*, *Nocardia*, *Propionibacterium*, *Pseudomonas*, or *Rhizobium*. To increase the efficiency of synthesis, precursors such as glycine, threonine, gamma-aminolevulinic acid, cobalt ions, or dimethylbenzimidazole are added to the medium.

Vitamin B12 deficiency can result in impaired hematopoiesis and neuronal function. Clinical symptoms of B12 deficiency include macrocytic anemia, neuropathies, affective changes in behaviour, and deterioration in cognitive function [15,16,17,18]. The Mayo Clinic gives a reference range for vitamin B12 in blood serum of 180–914 ng/dL [19], measured by competitive binding enzyme-linked immunosorbent assay. However, there is no standardized reference range or universal technique for measuring vitamin B12. In the United States and Australia, the range of vitamin B12 in human blood serum is 200–900 pg mL^−1^, and the cut-off for subclinical deficiency is 300 pg mL^−1^ [20,21].

Many analytical methods have been described for the quantitative determination of vitamin B12 in various biological matrices, such as dietary products and human serum samples [22], including the microbiological test using *Lactobacillus leichmannii* (ATCC 7830) [23], high-performance liquid chromatography (HPLC) with UV detection and electrochemical detection [24,25], fluorometry [26], inductively coupled plasma mass spectrometry (ICP-MS) [27], chemiluminescence (CL) assays [28], capillary electrophoresis (CE) [29], and surface plasmon resonance (SPR) biosensors [30].

Tsiminis, et al. [31] reviewed current methods dedicated to the measurement of vitamin B12 with a focus on optical spectroscopy, including chemiluminescence (CL) measurements, absorption and fluorescence spectroscopy, surface plasmon resonance (SPR), and Raman spectroscopy.

It should be emphasized that the techniques used for the detection and quantification of vitamin B12 differ significantly in detection sensitivity from the most sensitive, such as those based on radioisotopes and MS, for which the LOD is around 100 fg mL^−1^ [32]. Immunoassays, microbiological determination, and CL tests have LODs of 2.2, 20, and 890 pg mL^−1^, respectively [33,34,35]. Raman spectroscopy and HPLC have LODs of 250 ng mL^−1^ and 80 ng mL^−1^, respectively [36,37]. The least sensitive methods are CE, fluorescence, and SPR, with LOD values in the µg/mL range, namely 20 µg mL^−1^, 0.1 µg mL^−1^, and 1 µg mL^−1^, respectively [38,39,40].

However, for accurate quantification of an analyte present at trace levels in complex biological matrices, a selective separation and enrichment step is required to overcome matrix interferences and ensure sufficient detection sensitivity. To date, techniques such as solid phase extraction (SPE) [41] or dispersive solid phase extraction (d-SPE) or immunoaffinity extraction [42] have been used for sample preparation. In recent years, nanomaterials have become an increasingly used sorbent for the extraction of biomolecules in in vitro diagnostics. By appropriate adjustment of size, z-potential, and surface functionalization, nanomaterials enable preconcentration, selective separation, and ionisation analysis of proteins and metabolites in proteomics and metabolomics [43,44].

Biosensors based on nanomaterials with catalytic activity, called nanoenzymes, are also being developed for the rapid detection of clinical biomarkers [45]. Examples include Fe_3_O_4_ as a peroxidase mimetic (POD) [46], and noble metals and their oxides, metal–organic frameworks (MOFs), and carbon materials as nanoenzymes mimicking a variety of enzymes such as oxidase (OXD), superoxide dismutase (SOD), catalase (CAT), and others [47,48].

Magnetic solid phase extraction (MSPE) has many advantages over conventional methods. Thanks to the magnetic sorbent dispersed in the sample, the separation of the solid phase after adsorption of the analyte takes place using an external magnetic field without the need for centrifugation, user elution, or sample filtration. The dispersion provides an increased interfacial area between the sorbent and the sample, ensuring high adsorption capacity and short diffusion paths. [49,50].

Many examples of the use of magnetic nanoparticles for the thickening and removal of various organic and inorganic contaminants from complex environmental matrices have been described in the literature [51,52,53,54,55,56]. A common addition to the extraction mixture is ionic surfactants, which provide sorption of the analyte in the form of hemicelles. There is a report on the use of Fe_3_O_4_ modified with an anionic surfactant [sodium dodecyl sulphate (SDS)] for the concentration of vitamin B12 [4]. However, the search for suitable sorbents that would provide a simple, inexpensive, and environmentally friendly method of isolating and concentrating vitamin B12 from complex biological samples is still ongoing.

In this study, iron oxide magnetic nanoparticles (IONPs) were used for the dispersion, magnetic extraction, and initial enrichment of vitamin B12. Perfluorinated acids were used as an additive to ensure acidification of the environment and as an amphiphilic agent to allow sorption of the analytes of interest by the IONPs. Sorption conditions were optimized according to the composition of the extraction mixture and the time required to achieve effective sorption.

## 2. Results

### 2.1. Characteristics of IONPs

In our previous work, IONPs were characterized by scanning electron microscopy (SEM), energy dispersive spectrometry (EDS) analysis, X-ray photoelectron spectroscopy (XPS), as well as the Brunauer–Emmett–Teller (BET) and the DLS technique [57,58]. It has been found that IONPs obtained by co-precipitation belong to the mesoporous materials with a surface area (BET) of 151.4 m^2^ g^−1^, spherical shape, size ranging from 80 to 140 nm, and the presence of hydrous iron oxide FeO(OH) on the surface. The surface charge of the IONPs measured by DLS was negative, namely −7.24 ± 1.48 eV, in weak acidic conditions (pH = 6.66).

The nanoparticles obtained showed a characteristic magnetic response under the influence of an externally applied permanent magnet, which attracts the nanomaterial and separates it from the solution. However, this effect may also be the result of poor colloidal properties of the suspension, which is not stable in the dispersion medium [59].

**Figure 2:**
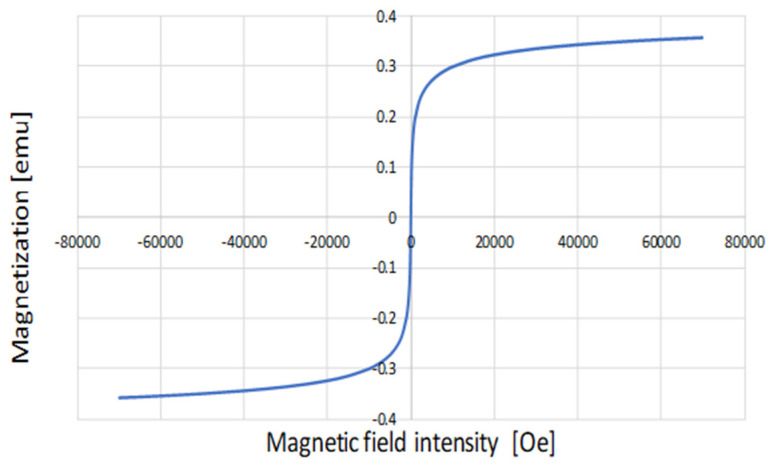
The hysteresis loops for IONPs measured at 300 K.

In order to characterize the magnetic behaviour of the obtained nanomaterial, the hysteresis loop was measured using a magnetometer. Figure 2 shows the magnetization curves of the powder sample at a temperature of 300 K. The hysteresis loop passed through the origin of the coordinate system, indicating no coercivity and no remanence, i.e., no residual magnetization. It can therefore be assumed that the sample analyzed is superparamagnetic and has an unstable magnetization. The saturation magnetization value (Ms) was 55.89 emu/g at 300 K, taking into account the mass of the sample, i.e., 6.38 mg, which is the basis for using a conventional magnet for magnetic phase separation.

### 2.2. Optimization of Cyanocobalamin Sorption by IONPs

**Figure 3:**
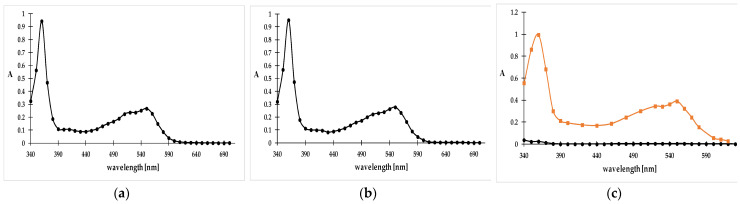
Spectrum of the aqueous solution of 0.05 mg mL^−1^ cyanocobalamin (**a**) after adding 75 mg of IONPs to 5 mL of the cyanocobalamin solution (**b**); the spectrum obtained after adding 75 mg of IONPs and 0.0588 g HFBA to 5 mL of the cyanocobalamin solution ((**c**), black); the spectrum obtained after adding 0.0588 g HFBA to 5 mL of 0.05 mg mL^−1^ cyanocobalamin solution ((**c**), orange).

Figure 3a–c illustrate the change in absorption intensity of the aqueous solution of cyanocobalamin after treatment with IONPs alone and IONPs with heptafluorobutanoic acid (HFBA). Cyanocobalamin is characterized by two strong absorption bands at 360 nm and 550 nm. It can be seen that after 30 min contact time with IONPs, the intensity of the absorption peaks did not change. A decrease in absorption was observed after the addition of HFBA to this mixture. The changes in the spectrum were accompanied by the disappearance of the pink colour of the cyanocobalamin solution. A similar effect was observed after the addition of another additive such as TCAA. Therefore, cyanocobalamin sorption only occurs in the presence of perfluorinated acids, which provide an acidic environment and an anion with ion-pair properties. The addition of the acid per se did not change the cyanocobalamin spectrum, as can be seen in Figure 3c. Thus, the changes in absorption intensity were due to the adsorption process and not to cyanocobalamin denaturing or a chemical reaction caused by the effect of the acid. TFAA was excluded from further testing as dissolution of the nanoparticle was observed in the presence of this acid.

**Figure 4:**
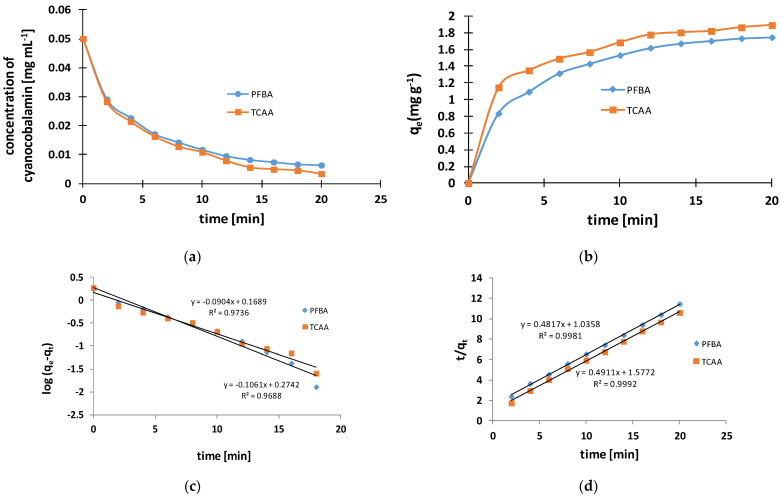
Kinetics of cyanocobalamin adsorption on IONPs. (**a**) Effect of contact time on measured concentration of analyte in mg mL^−1^; (**b**) effect of contact time on the amounts of cyanocobalamin adsorbed per mass of adsorbent (g) at equilibrium in mg g^−1^; (**c**) pseudo-1st-order and (**d**) pseudo-2nd-order models. Experimental conditions: cyanocobalamin (0.05 mg mL^−1^) and IONP dosage (0.075 g).

In order to optimize the sorption conditions, the influence of the mass of HFBA/TCAA, the mass of IONPs, and the time necessary to isolate cyanocobalamin from the solution were examined. The effect of contact time on the adsorption of cyanocobalamin onto IONPs is shown in Figure 4. As can be seen in the graphs in Figure 4a,b, the adsorption capacity of cyanocobalamin by IONPs increases systematically within 20 min and then reaches a plateau, therefore extending the time may not be as effective due to the saturation of the available adsorption sites. To investigate the adsorption mechanism, the kinetic data were fitted to pseudo-first- and pseudo-second-order models (Figure 4c,d).

Pseudo first order model:

(1) $$\ln\left(q_{e}-q_{t}\right)=lnq_{e}-k_{1}t$$

Pseudo second order model:

(2) $$\frac{t}{q_{t}}=\frac{1}{k_{2}q_{e}^{2}}+\frac{t}{q_{e}}$$

where *q_e_* and *q_t_* are the mass of cyanocobalamin in mg adsorbed per g of IONPs at time *t* and at equilibrium, and *k*_1_ and *k*_2_ are the constants of the pseudo first and second order models, respectively. The *q_e_* value was calculated according to the following equation:

(3) $$q_{e}=\frac{{(c}_{0}-c_{e})V}{m}$$

where *c*_0_ and *c_e_* are the cyanocobalamin initial and equilibrium state concentrations, respectively, *V* is the sample volume, and m is the mass of IONPs.

The kinetic parameters for the above models are summarized in Table 1. As can be seen, both models showed a very good fit to both models. However, a higher value of the determination coefficient *R*^2^ was obtained for the pseudo-2nd-order model for the initial cyanocobalamin concentration of 0.05 mg mL^−1^.

**Figure 5:**
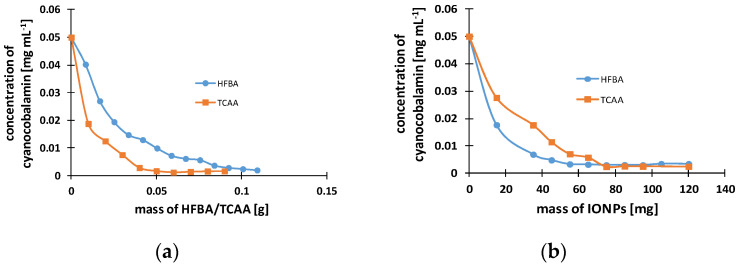
The experimental curves representing the adsorption kinetics of cyanocobalamin into the IONPs. Analysis conditions: solution volume 3 mL, spectrophotometric measurement at a wavelength of 360 nm, initial concentration of cyanocobalamin 0.05 mg mL^−1^, mass of IONPs 75 mg (**a**), 0.1259 g HFBA/0.06 g TCAA (**b**).

The influence of mass of IONPs and acidic additives on the adsorption of cyanocobalamin onto the IONPs is demonstrated in Figure 5. The graphs show that both acids, PFBA and TCAA, provide similar sorption kinetics of cyanocobalamin on IONPs. However, Figure 5a indicates that TCAA is more effective in the sorption of cyanocobalamin. A total of 0.12 g of HFBA removes 96% of cyanocobalamin, while to remove this amount of cyanocobalamin, half the amount of TCAA is needed, i.e., 0.06 g. Trifluoroacetic acid (TFAA) turned out to be unsuitable because after its addition to the extraction mixture, a color change from pink to orange–brown occurred. Therefore, further experiments with TFAA were discontinued. The effect of IONP mass on the adsorption of cyanocobalamin was also examined (Figure 5b). As can be seen in the graph, the IONP mass of 0.075 g ensures the adsorption of cyanocobalamin at a concentration of 0.05 mg mL^−1^. After 0.075 g of IONPs, we observe a plateau, which confirms the fact that this mass of IONPs was enough to remove 0.05 mg mL^−1^ of cyanocobalamin.

### 2.3. Adsorption Isotherms

**Figure 6:**
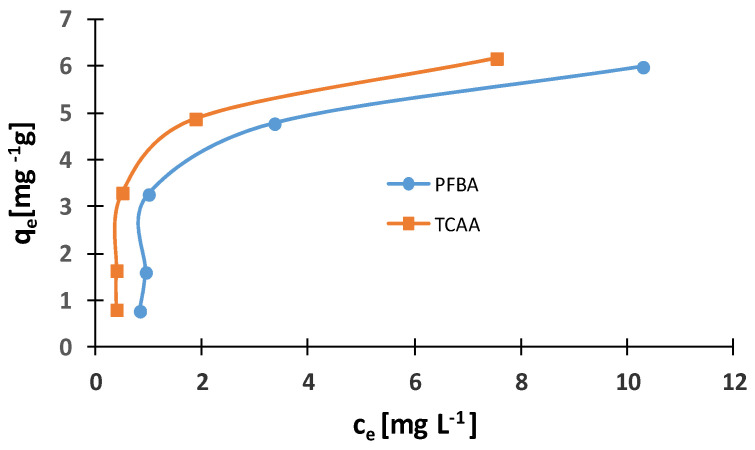
Isothermal adsorption of cyanocobalamin on IONPs. Experimental conditions: contact time (20 min); temperature (r.t); cyanocobalamin (0.05 mg mL^−1^) and IONPs dosage (0.075 g), 0.06 g HFBA/TCAA.

The adsorption isotherms are the basic relations enabling description of the properties of sorption materials and evaluate their usefulness, which is indispensable while designing adsorption systems. The determined experimental amounts of vitamin B_12_ (*q_e_*), depending on their equilibrium concentration in the solution (*C_e_*), are presented in Figure 6. As can be seen, the adsorbed amount of cyanocobalamin increases with increasing equilibrium concentration (*c_e_*). The maximum adsorption capacity exceeds 6 mg g^−1^. The sorption process can be described with Freundlich and Langmuir equations. The values of parameters for Langmuir and Freundlich isotherms can be recorded using a linear regression analysis from the following linear expressions, respectively:

(4) $$\frac{c_{e}}{q_{e}}\;vs\;c_{e}$$

(5) $$lnq_{e}\;vs\;lnc_{e}$$

The values of parameters for Langmuir and Freundlich isotherms are presented in Table 1.

Freundlich isotherms assume non-ideal and multi-layer adsorption of a solute on a heterogeneous adsorbent surface. The data collected in the Table 2 for the Freundlich isotherm have *R*^2^ values greater than 0.6 and describe the examined case quite poorly. The Langmuir isotherm describes the adsorption data slightly better with *R*^2^ values between 0.7–0.9. The fact that the tested system favors the description of adsorption using the Langmuir isotherm is also supported by the fact that the maximum adsorption capacity *q_m_* (8.9; 7.7 mg g^−1^) calculated on the basis of the Langmuir isotherm (Table 2) is close to the experimental value *q_e_* of cyanocobalamin adsorption on IONPs. This suggests rather homogeneous adsorption of solute onto the adsorbent surface. Moreover, we can assume single-layer adsorption as the upper adsorption limit. The Langmuir isotherm can be characterized by a dimensionless constant which is called also separation factor (*R_L_*), defined as: *R_L_* = 1/(1 + *K_L_c*_0_), where: *c*_0_—initial concentration of analyte in the solution (mg/dm^3^); *K_L_*—the Langmuir isotherm constant (dm^3^/mg) [60]. The separation factor indicates whether the adsorption is favorable (0 < *R_L_* < 1) or not (*R_L_* > 1), where *R_L_* = 1 (the type of isotherm is linear) [61]. In the tested case, the *R_L_* value is greater than 0 and less than 1 and is exactly 0.083 and 0.032 in the case of the addition of PFAAs and TCAAs, respectively, which confirms the fact that adsorption is preferred under steady conditions. Chemisorption is likely to occur and the interactions between the adsorbed molecules are very small [62,63]. Therefore, the inclusion of two isotherm models for analytical adsorption may indicate that single-layer and heterogeneous surfaces participate in the adsorption process.

### 2.4. Selectivity of the Sorption onto IONPs

**Figure 7:**
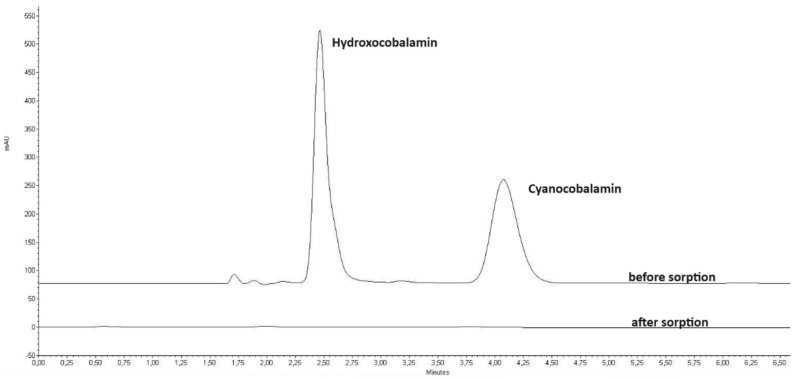
Chromatogram of the mixture of hydroxy-and cyano-cobalamin before and after the sorption experiment conducted using 75 mg of IONPs, 0.06 g of TCAA, 5 mL of 0.05 mg mL^−1^ cyanocobalamin solution, with an extraction time of 20 min. Chromatographic conditions: eluent: 30% methanol with 0.025%TFA, column: Zorbax Extend C18, detection wavelength: 360 nm.

In order to verify the selectivity of cyanocobalamin sorption by IONP, the analysis was carried out in the presence of its hydroxy form, which has a hydroxyl instead of a cyano group bound to cobalt in the trivalent state, Co(III). This study was carried out under optimal experimental conditions with a constant concentration of both forms of 0.05 mg mL^−1^. The results are shown in the chromatograms in Figure 7. As can be seen, the above forms of cobalamin are sorbed simultaneously onto the IONP. The lack of selectivity shows that the proposed procedure ensures the sorption of different forms of cobalamin by IONPs.

### 2.5. Desorption Conditions

**Figure 8:**
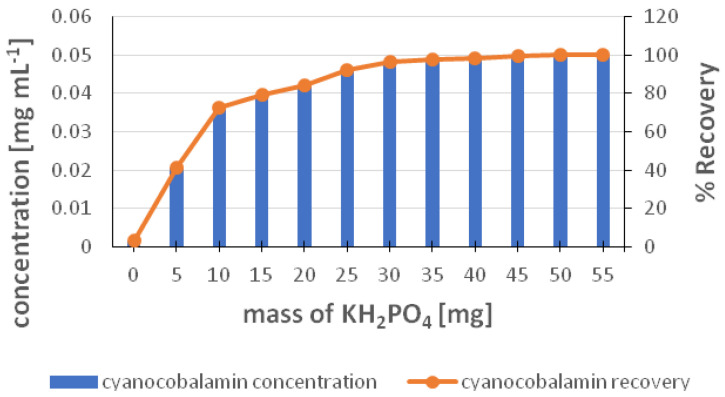
Desorption cycles of cyanocobalamin after sorption onto IONP adsorbent. Sorption conditions: concentration (0.05 mg mL^−1^), TCAA additive (0.06 g); IONP dosage (75 mg). Desorption conditions: sample volume (5 mL); contact time (15 min); mass of KH_2_PO_4_ in the range of 5–55 mg.

To estimate the desorption of cyanocobalamin from the surface of IONPs, the addition of a saline solution was chosen. Since sorption takes place at an acidic pH, a higher pH was chosen for desorption, which favors the breaking of the pair of anionic additive and target analyte. As can be seen in Figure 8, which shows the dependence of the % desorption of cyanocobalamin on the mass of KH_2_PO_4_, the addition of 50 mg of salt ensures 100% efficiency of the desorption process. The effect of the desorption time on the recovery of cyanocobalamin was also tested, but there was no significant effect when the desorption time was longer than 15 min. Therefore, for the following experiments, a time of 15 min was chosen as sufficient for the quantitative desorption of cyanocobalamin from IONPs.

The long-term stability of adsorbed cyanocobalamin on IONPs was also investigated, which could be crucial for storage and subsequent analysis. For this purpose, 5 samples were prepared according to previously optimized conditions, i.e., 75 mg IONPs, 0.6 g TCAA, 5 mL 0.05 mg mL^−1^ cyanocobalamins, and 20 min incubation. The modified NPs were magnetically separated and stored at room temperature away from light. In the following days, further samples were desorbed using 50 mg KH_2_PO_4_ dissolved in 5 mL of water and the percentage recovery of cyanocobalamin was calculated. The recoveries obtained in the following days were as follows 101.2%, 100.8%, 103.4%, 101.65%, and 100.62%, indicating the stability of the adsorbed cyanocobalamin on the nanoparticles. It is therefore possible to store the modified nanoparticles at room temperature for several days for further analysis or other applications.

### 2.6. FT-IR/ATR Analysis

**Figure 9:**
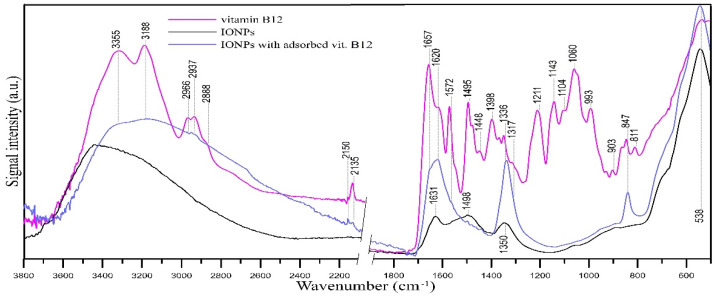
FT-IR/ATR spectra of vitamin B12 (magenta line), IONPs (black line), and IONPs with adsorbed vitamin B12 (blue line).

FT-IR/ATR spectroscopy was used to confirm the sorption of vitamin B12 on IONPs. The samples were air-dried before IR measurements, because water itself causes the presence of intense and broad bands in the IR spectrum. The use of mild drying conditions ensured that no decomposition of the adsorbed vitamin B12 occurred. The spectra are shown in Figure 9.

Analysis of the bands in the spectrum of pristine IONPs (Figure 9, black line) indicates the presence of iron in the form of oxide [64]. The major bands visible in the vitamin B12 spectrum (magenta line) are due to the asymmetric (3316 cm^−1^) and symmetric (3188 cm^−1^) N-H stretching in amide I, aliphatic C-H stretching within 2970–2870 cm^−1^, cyanide CN stretching vibrations at 2135 cm^−1^, C=O stretching in amide I of the propionamide side chains of the corrin ring at 1657 cm^−1^, breathing mode of the corrin ring at 1572 cm^−1^ [65,66], C-H deformation vibration at 1398 and 1336 cm^−1^, C-O stretching vibration at 1211, 1143 and ~1020 cm^−1^ [67], and PO_4_^3−^ in phosphate groups at 1060 and 993 cm^−1^. In the spectrum of IONPs after adsorption of vitamin B12 (Figure 9, blue line), new bands can be observed, not present in the IR spectrum of pristine IONPs. There are changes in the intensity and position of the bands in the range of 3355–3188 cm^−1^. The bands responsible for the presence of N-H group vibrations (3355, 3188 cm^−1^) and low intensity bands of C-H group vibrations (1336 cm^−1^, ~2940 cm^−1^) appear as well as bands indicating the presence of amide groups of adsorbed vitamin B12 (shoulder at ~1657 cm^−1^ and intense band at 1620 cm^−1^). Evidence for the vitamin B12 sorption onto IONPs is the presence of a low-intensity band of CN groups at 2135 cm^−1^. Additionally, this band is slightly shifted towards higher wavenumbers (up to ~2150 cm^−1^), which may indicate a weakening of the cobalt-CN bond in vitamin B12 upon binding to the IONPs. Since the sorption of vitamin B12 took place in the presence of trichloroacetic acid, bands of the latter can also be expected in the FT-IR spectrum: its presence on the surface of IONPs may be indicated by the band at 847 cm^−1^ (C-Cl). The band at 1336 cm^−1^ may be caused by both the presence of deformation vibrations of -OH in carboxyl groups and the presence of deformation vibrations of CH_2_ groups in vitamin B12 molecules. Similarly, the band at 1620 cm^−1^ may also have a contribution from trichloroacetic acid (C=O symmetric stretching in -COOH) [68].

### 2.7. Application of the Spectrophotometric Method for the Determination of Cyanocobalamin in Urine Sample

The calibration curve was constructed from seven standard solutions by plotting the absorbance against the nominal concentration of the standard. The linear seven-point calibration curve was obtained at concentrations ranging from 0.005 to 0.1 mg mL^−1^ (0.005; 0.01; 0.015; 0.025; 0.05; 0.08; 0.1 mg mL^−1^). Quantitative determination was based on a standard curve or comparison with the absorbance of the standard solution at a given concentration within the linear responses.

The recovery and repeatability of the developed isolation method were determined by spiking urine with cyanocobalamin standard solutions of low, medium, and high levels of the analytical concentration range for three replicates. A total of 1 mL of the urine sample was spiked with 8, 40, and 90 µg mL^−1^ cyanocobalamin and treated according to the recommended procedure. The recoveries ranged from 101.47% to 107.99% (Table 3) and the relative standard deviation of repeatability (RSDr) ranged from 1.25% for the highest to 5.43% for the lowest cyanocobalamin content in the sample analyzed.

## 3. Discussion

Vitamins are essential for proper growth and development of the body. They perform a variety of functions in the body, including: coenzymes, antioxidants, regulators of genetic processes. Vitamins are manufactured on an industrial scale for the dietary supplements, cosmetics, and food and feed fortification markets.

In the case of vitamin B12, its content in samples of various origins is determined after cyanidation to cyanocobalamin during sample preparation. The efficacy of cyanidation conversion has been confirmed in a 2023 study [69]. This approach is preferred because it reduces the number of analytes assayed for CNCbl, increases the analyte concentration, and improves analyte stability.

One of the analytical difficulties in the determination of vitamin B12 is the adjustment of the sensitivity of methods that would allow the determination of trace amounts of this vitamin in samples. Sensitive and advanced analytical techniques exist for the quantification of cyanocobalamin, such as mass spectrometry, fluorimetry, and anodic adsorption voltammetry, but in practice liquid chromatography (HPLC) is the most widely used. The limitation of HPLC for the determination of traces of cyanocobalamin is the relatively high limit of detection (LOD) and limit of quantification (LOQ). The parameters of the cyanocobalamin calibration curve obtained by HPLC using the DAD and FL detection modes were compared with those obtained by spectrophotometry (Table 4).

**Figure 10:**
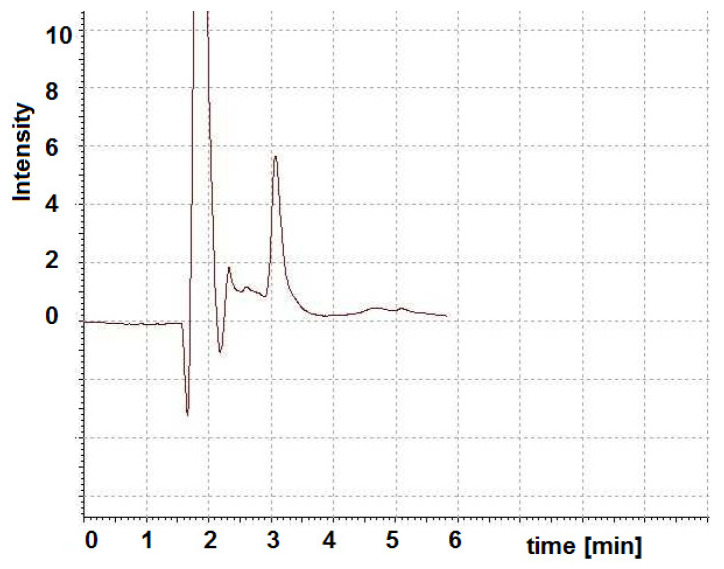
Representative HPLC-DAD chromatograms of blank samples prepared according to the proposed procedure.

The parameters collected in Table 4 prove that the most favorable LOD and LOQ are provided by HPLC with FL detection. In the case of the DAD detector, calibrations were made for two wavelengths, i.e., 218 and 360 nm, corresponding to the absorption bands of cyanocobalamin. Both selected wavelengths are outside the cut-off range for the eluent components, i.e., methanol (cut-off value 205 nm), water (cut-off value 190 nm), and trifluoroacetic acid (cut-off value 210 nm). Detection in the ultraviolet range turned out to be more advantageous in terms of LOD and LOQ units. However, it should be remembered that in the case of low-quality solvents, a signal from the solvent may appear which will overlap with the signal of the substance, which will lead to incorrect fractionation. Other interferences appearing in the chromatogram at a retention time of approximately 3 min, i.e., the retention time of cyanocobalamin, include peaks due to negative phosphate anions, which in the proposed method serve to desorb cyanocobalamin from nanoparticles (Figure 10). FL detection provides greater sensitivity and does not interfere with the retention of the analyte being tested.

The most unfavorable LOD and LOQ limits at the µg mL^−1^ level were obtained for the spectrophotometric measurements. The limitations of low detection sensitivity can be overcome by using appropriate separation and preconcentration techniques. When it comes to sample preparation, methods are sought that are simple, cost-effective, environmentally friendly, and effective in eliminating adverse matrix effects while maintaining a low initial analyte concentration. Recently, magnetic solid phase extraction (MSPE) has become increasingly popular [70]. Magnetic sorption nanomaterials have a larger contact surface, sorption efficiency, and selectivity. Not without significance is the fact that they do not require special laboratory tools, as extraction can be performed in batch mode, and phase separation can be performed using an external magnet.

Nanoparticles were previously used to isolate cyanocobalamin from biological fluids such as urine. An example is the work describing the use of silver nanoparticles embedded in chitosan for this purpose, followed by extraction to the cloud point using Triton X-100, and fluorescence detection [71]. In this study, the calibration curve was linear in the range of 0.2–15.0 ng mL^−1^, the LOD and LOQ were 0.036 and 0.119 ng mL^−1^, respectively. In another study [4], vitamin B12 was extracted from pharmaceutical preparations using Fe_3_O_4_ magnetic nanoparticles (MSPE) modified with sodium dodecyl sulfate (SDS). The vitamin was desorbed with alkaline 1-propanol. Quantitative analysis was performed using flow injection inductively coupled plasma–optical emission spectrometry. The linear range of the method was 2.5–500 µg L^−1^ and the LOD was 1.0 µg L^−1^.

The proposed procedure ensures effective sorption of cyanocobalamin from aqueous solutions at the level of 8.9 or 7.7 mg g^−1^ of IONPs depending on the added acid (HFBA or TCAA) and effective desorption with an aqueous solution of potassium dihydrogen phosphate. For comparison, in the work of Lupaşcu, et al. [72], the sorption of vitamin B12 by mesoporous activated carbons obtained from lignocellulosic plant raw materials, i.e., nut shells and apple wood, was investigated. The authors of the study emphasize that the proposed carbon sorbents are effective for immobilization of vitamin B12 and creatinine with the maximum adsorption of vitamin B12 ranging between 238.77 mg·g^−1^ and 295.54 mg·g^−1^. However, the authors propose the use of these activated carbon sorbents as enterosorbents to detoxify the body from endogenous and exogenous toxins.

The usefulness of the developed method was tested in the biological matrix of human urine. Taking into account the limitations discussed above, a spectrophotometric method was chosen for the quantitative determination of cyanocobalamin, which, despite less favorable LOD and LOQ, does not require the use of advanced analytical equipment and can be performed in most laboratories. In addition, the sample matrix after desorption does not affect the result, especially when used as a blank in measurements. The low LOD and LOQ limits found for spectrophotometric measurements can be overcome by using larger sample volumes, which is not a problem for urine, which can be collected in larger volumes and by non-invasive methods.

To date, very sensitive detection methods have been used to determine cyanocobalamin in body fluids, i.e., blood serum or urine, such as high-performance liquid chromatography/inductively coupled plasma mass spectrometry (HPLC/ICP-MS), which has an LOD of 0.05 ng mL^−1^ [73]. In 2009, Mandal et al. [74] described a method for the determination of cyanocobalamin in urine using HPLC with recoveries of 90% and 95.8% for concentrations of 2 and 12 µg mL^−1^, respectively. The authors demonstrated linearity over the concentration range of 2–16 µg mL^−1^, the limit of detection (LOD) was 0.04 µg mL^−1^, and urinary cobalamin levels varied from 0.04–0.2 µg mL^−1^. In 2012, Berton et al. [75] described the selective extraction of vitamin B12 in urine by ABS (aqueous two-phase system) based on ionic liquids before HPLC. The authors achieved 97% extraction efficiency by spiking samples with 1 and 3 µg mL^−1^, an LOD of 0.09 µg mL^−1^, and a linear range of 0.40–8.00 µg mL^−1^. It should be noted that in methods using liquid-solid/liquid extraction, the recoveries obtained vary widely, from 65–130% when immunoaffinity cartridge (IAC) is used for sample preparation [76,77], 97–106% for ionic liquid-based aqueous two-phase system (ATPS) [75], 86–96% in the case of SPE [78], and even 46% for MNPs-based SPE applied for cyanocobalamin isolation [4]. In comparison, the recovery of the current study was in the range of 101–107% at three concentration levels, i.e., 8, 40, and 90 µg mL^−1^ with RSD from 5.43 to 1.25%.

The proposed method for the isolation and determination of cyanocobalamin is simple and inexpensive and does not require sophisticated equipment. A total of 1 g of IONPs absorbs about 8 mg of cyanocobalamin from an aqueous solution and, thanks to the magnetic properties of the nanoparticles, it can be easily separated from the solution and concentrated to a small volume, ensuring the concentration of the analyte. In comparison, the sorption capacity of an immunoaffinity column is limited to about 1 µg per column [79], whereas magnetic Fe_3_O_4_ nanoparticles modified by sodium dodecyl sulfate (SDS) synthesized by Yamini et al. [4] absorb 100 µg of cyanocobalamin per 40 mg NPs, which is the equivalent of 2.5 mg/g. The isolated cyanocobalamin can be further quantified using different techniques with different sensitivities (HPLC-DAD, HPLC-FL, spectrophotometry, etc.). It should be noted, however, that after desorption the sample contains KH_2_PO_4_, which may be a source of the interferences in HPLC-DAD.

The use of HFBA, which belongs to the group of perfluoroalkyl substances (PFAS), as an additive to the sorbent mixture may raise some concerns. These compounds are considered toxic and persistent in the environment due to the high strength of the carbon-fluorine bond. However, it is the long-chain PFAS such as perfluorooctanoic acid (PFOA) and perfluorooctane sulphate (PFOS) that are of concern. Short-chain PFASs have long been considered safe because they have much shorter half-lives [80]. According to 2018 data, the half-life of HFBA is hours in mice and several days in humans, which seems to be a favorable alternative compared to the long half-lives of FOA and PFOS [81]. Animal toxicity studies of HFBA have shown effects on cholesterol levels [82], thyroid function, liver function, and reproduction [83]. Little is known about human exposure to HFBA, although this chemical has been detected in water [84] and consumer products [85]. According to recent studies in a mouse model, HFBA absorbed through the skin causes similar toxic effects in the liver as oral exposure [86]. Therefore, dermal exposure to HFBA should be avoided due to the potential for skin penetration and health risks.

The mechanism responsible for the sorption of cyanocobalamin onto IONPs should be the subject of further research. Future considerations should take into account the possibility of forming hemimicelles, mixed hemimicelles, and admicelles on the surface of nanoparticles, analogously to the adsorption of a surfactant [52]. Another possibility of interpretation includes the so-called chaotropic effect widely discussed in chromatography [87,88]. In short, perfluoric acids belonging to the so-called chaotropic ions disrupt the solvation zone of the solvated ionic analyte, increasing its hydrophobicity. A zeta-potential isotherm study can be applied to demonstrate the adsorption of amphiphilic substances and for the identification of the types of aggregates formed.

## 4. Materials and Methods

### 4.1. Materials

Cyanocobalamin (vitamin B_12_, vit. B_12_) was purchased from Sigma-Aldrich Inc., St. Louis, MO, USA. FeCl_3_ × 6H_2_O, FeSO_4_ × 4H_2_O, and potassium dihydrogen phosphate (KH_2_PO_4_) were purchased from Merck (Darmstadt, Germany). Ammonia solution (25% wt.%) was obtained from POCH S. A. (Gliwice, Poland). Methanol was purchased from E. Merck (Darmstadt, Germany). Trifluoroacetic acid (TFAA) suitable for HPLC, ≥99.0%, trichloroacetic acid (TCAA) ACS reagent, ≥99.0%, and heptafluorobutyric acid (HFBA, perfluorobutyric acid PFBA), ≥99.5% were purchased from Sigma-Aldrich Inc., St. Louis, MO, USA. Water with a resistivity of 18.2 MΩ cm was obtained from an ULTRAPURE Millipore Direct-Q 3UV-R (Merck, Darmstadt, Germany).

### 4.2. Synthesis of Iron Oxide Nanoparticles (IONPs)

The synthesis of IONPs was performed using the co-precipitation method. A total of 20 mL of 25% ammonia aqueous solution was added dropwise to a 100 mL solution containing a mixture of iron salts in 2:1 molar ratio of Fe(III) to Fe(II) (2.703 g FeCl_3_ × 6H_2_O, 1.120 g FeSO_4_ × 4H_2_O) with constant stirring at a room temperature. The reaction medium quickly changed color from orange to black, indicating the formation of Fe_3_O_4_ nanoparticles. Stirring was continued for another hour. The obtained precipitate was separated from the reaction solution using a magnetic field, and washed several times with deionized water and dried.

### 4.3. UV-VIS Spectroscopy

A Genesys 10S UV-VIS spectrophotometer (Thermo Fischer Scientific, Madison, WI, USA) was used for the absorbance measurements. The cyanocobalamin quantification was performed at 361 nm.

### 4.4. HPLC-DAD

The HPLC-DAD system VWR/Hitachi LaChrom Elite (Merck, Rahway, NJ, USA) equipped with the LaChrom Elite L-2455 DAD detector, LaChrom Elite L-2485 fluorescence detector, a manual injection valve, and a thermostat for chromatographic columns was used. As the stationary phase, a Zorbax Extend C18 Agilent Technologies (Santa Clara, CA, USA) column (150 mm × 4.6 mm I.D., 5 μm) was utilized for chromatographic analysis. A concentration of 0.025% trifluoroacetic acid (TFAA) in 30% methanol served as a mobile phase.

### 4.5. Sorption Isotherms

A batch equilibrium experiment was conducted using cyanocobalamin concentrations in a solution ranging from 12.5 to 100 µg L^−1^ as follows: 75 mg of IONPs were equilibrated with 5 mL of solutions containing 12.5, 25, 50, 75, and 100 mg mL^−1^ in centrifuge tubes for 24 h on a reciprocating shaker at room temperature. After equilibration the phases were separated by magnet. Two replicates were used for collecting each data point. Cyanocobalamin concentrations in the supernatant were measured spectrophotometrically. The amount of sorbed analyte was calculated as the difference between the initial and final concentration. The obtained data was fitted to Langmuir and Freundlich isotherms.

Cyanocobalamin sorption data were fitted to Freundlich equation using the formula: *q* = *k_f_* *c^n^*, where: *q* is the sorbed analyte amount in mg kg^−1^; *c* is the equilibrium solution concentration in mg L^−1^. The model was linearized by using the logarithmic transformation resulting in the predictive equation log (*q*) = log (*K_f_*) + *n* log(*c*). Estimates of *K_f_* were obtained using exp (log (*K_f_*)). The distribution coefficient (*K_d_*) values were calculated according to the formula: *K_d_* = *q*/*c* = *k_f_* *c^n^*/*c* = *k_f_* *c*^*n*−1^ [89,90].

The Langmuir equation has the following form: *c_e_*/*q_e_* = 1/*q_m_K_L_* + *c_e_*/*q_m_*, where: *c_e_*—the equilibrium concentration of analyte in solution (mg/dm^3^); *q_e_*—the amount of the analyte sorbed per unit mass of the IONPs (mg g^−1^); *q_m_*—the maximum amount of vitamin B_12_ covering the surface of the nanoparticles (monolayer capacity) (mg g^−1^); *K_L_*—the Langmuir isotherm constant (dm^3^ mg^−1^) [62].

### 4.6. FT-IR/ATR Analysis

Attenuated total reflectance Fourier transform infrared (FT-IR/ATR) spectra dried samples (air drying, room temperature, 24 h) were recorded in the 3800–500 cm^−1^ range, resolution 4 cm^−1^, at room temperature using Nicolet 6700 spectrometer and Meridian Diamond ATR accessory (Harrick, Pleasantville, NY, USA). Samples (cyanocobalamin standard, synthesized IONPs and modified IONPs under optimal conditions, namely a mixture of 75 mg IONPs, 0.06 g TCAA and 5 mL of 0.05 mg mL^−1^ cyanocobalamin solution, were separated into solid and liquid phases by an external magnet after 20 min mixing, then the liquid phase was discarded and the solid part was dried for further analysis) were directly applied onto the diamond crystal, and close contact was made with the surface by a pressure tower. The spectrum consisted of 256 scans. Dry potassium bromide (48 h, 105 °C) was used to collect background spectrum. All spectra were corrected for water vapor and carbon dioxide and ATR correction was applied. No smoothing functions were applied. All spectral measurements were performed at least in triplicate.

### 4.7. Magnetic Measurements

The magnetic measurements were performed using a Superconducting Quantum Interference Device (SQUID) magnetometer MPMS-3 (Magnetic Property Measurement System from Quantum Design, Inc., San Diego, CA, USA) at a constant temperature of 300 K.

### 4.8. DLS Measurements

The ζ-potential of the IONPs were evaluated using a dynamic light-scattering (DLS) particle-size analyzer (Zetasizer Nano ZS; Malvern Instruments, Malvern, UK).

### 4.9. The Recommended Procedure for Recovery of Cyanocobalamin from the Urine Sample

The urine sample was collected from a volunteer who gave written informed consent to provide the material for these tests. The urine was subjected to routine laboratory analysis, which showed no abnormalities (pH = 6.5; creatinine 0.68 mg/dl; glucose 85 mg/dl; folic acid 11.4 ng/mL; eGFR > 90 mL/min/1.73 m^2^). Morning urine samples were collected into clean vials, and centrifuged for 5 min at 5000 g to remove any possible insoluble precipitates. The supernatant was transferred to a 20 mL vial and immediately analyzed. The urine samples were spiked with three concentration levels of cyanocobalamin and analyzed by the recommended procedure. IONPs in the amounts of 75 mg, and 60 mg TCAA were added to 1 mL of spiked urine samples provided from volunteers. The samples were shaken for 20 min using a Bio RS-24 Mini Rotator (BioSan, Medical-biological Research and Technologies, Riga, Latvia) at 30 rpm vertical rotation movement (360°). The experiment was conducted in three parallel repetitions. Then, the supernatant was removed. Desorption of the analyte was performed by shaking for 15 min with 2 mL of an aqueous solution containing 55 mg of KH_2_PO_4_. Analyte concentrations were measured spectrophotometrically at 361 nm against an unspiked urine sample analyzed by the same procedure as a blank sample. The percent of recovery (%*R*) was calculated using the following formula: %*R* = (*c_f_* − *c*_0_)/*c*_0_ 100%, where: *c*_0_ and *c_f_*—concentration of the analyte added and found after extraction, respectively.

## 5. Conclusions

The conducted research confirmed the usefulness of mesoporous superparamagnetic nanoparticles, or IONPs, for the selective sorption of cyanocobalamin from aqueous solutions in the presence of perfluorinated acids, especially TCAA and HFBA. The proposed procedure based on IONPs is simple, cheap, and effective. Kinetic measurements showed that the maximum values of adsorption capacity obtained in the Langmuir model were consistent with experimental data. Spectrophotometric determination of trace amounts of cyanocobalamin is possible despite the relatively high LOD value. In this case, a larger sample volume is required to recover a larger mass of analyte. Furthermore, the adsorbed cyanocobalamin on the nanoparticles is stable; it is, therefore, possible to store the modified nanoparticles at room temperature for several days for further analysis or other applications.

## Figures and Tables

**Table 1:** Kinetic parameters of cyanocobalamin adsorption by IONPs.

Kinetic Model	Parameter	HFBA	TCAA
Pseudo-1st-order	*q_e_* mg g^−1^	1.3200	1.1800
	*k*_1_ min^−1^	0.0904	0.1061
	*R* ^2^	0.9688	0.9736
Pseudo-2nd-order	*q_e_* mg g^−1^	2.0760	2.0360
	*k*_2_ mg g^−1^ min^−1^	0.2240	0.4912
	*R* ^2^	0.9981	0.9992

Experimental conditions: cyanocobalamin 0.05 mg mL^−1^, IONP dosage 75 mg, 0.06 g HFBA or TCAA.

**Table 2:** The values of parameters for Langmuir and Freundlich isotherms.

Isotherm Type	Coefficient	Unit	HFBA	TCAA
Freundlich	*n*	range from 0 to 1	1.0469	0.5236
	*K_f_*	(mg g^−1^)/(L mg^−1^)^n^	0.7158	2.6203
	*R* ^2^	-	0.6472	0.6577
Langmuir	*q_m_*	mg g^−1^	8.9047	7.6923
	*K_L_*	L mg^−1^	0.2203	0.5996
	*R* ^2^	-	0.6982	0.899

**Table 3:** The recoveries of cyanocobalamin from the artificial urine samples.

Vitamin B12 Added	Vitamin B12 Found	Relative Error [%]	Recovery [%]	RSD [%]
8.00 µg mL^−1^	8.64 µg mL^−1^	8.0	107.99	5.43
40.00 µg mL^−1^	40.59 µg mL^−1^	1.5	101.47	1.25
90.00 µg mL^−1^	91.43 µg mL^−1^	1.6	101.59	1.80

**Table 4:** The linear regression function parameters obtained for calibration curves prepared for cyanocobalamin quantification using chromatographic and spectrophotometric techniques.

Parameter	DAD Detection [360 nm]	DAD Detection [218 nm]	FL Detection λ_ex_ = 250 nm, λ_em_ = 360 nm	Spectrophotometry λ_em_ = 361 nm
linearity range	0.39–100 μg mL^−1^	1.56–50 μg mL^−1^	95.35 ng mL^−1^–25 μg mL^−1^	5–100 μg mL^−1^
calibration equation	Y = 86,725 (±1353) + 146,879 (±52100)	y= 129,767 (±5846) + 111,312 (±137,786)	y = 78,930,496 (±1,518,746) + 858,972 (±17,146)	y = 18.195 (±0.297)x + 0.0148 (±0.015)
correlation coefficient	0.9983	0.9919	0.9989	0.9987
S_e_	127,744	242,297	32,458	0.027
F	4104	492	2701	3741
LOD ^1^	72.97 ng mL^−1^	26.86 ng mL^−1^	12.89 ng mL^−1^	2.64 μg mL^−1^
LOQ ^1^	243.24 ng mL^−1^	80.58 ng mL^−1^	42.95 ng mL^−1^	8.24 μg mL^−1^

^1^ The LOD value was calculated at a signal-to-noise (S/N) ratio of 3; the LOQ value was calculated at a signal-to-noise (S/N) ratio of 10 in the case of chromatography, and LOD = 3 σ/slope, LOQ = 10 σ/slope in case of spectrophotometry. The calibration curves were obtained by plotting the concentration (x, µg mL^−1^), versus chromatographic peak area (y) or the absorption value for spectrophotometry.

## Data Availability

Data will be made available on request from J. Flieger.
